# Disparities by Race and Ethnicity Among Adults Recruited for a Preclinical Alzheimer Disease Trial

**DOI:** 10.1001/jamanetworkopen.2021.14364

**Published:** 2021-07-06

**Authors:** Rema Raman, Yakeel T. Quiroz, Oliver Langford, Jiyoon Choi, Marina Ritchie, Morgan Baumgartner, Dorene Rentz, Neelum T. Aggarwal, Paul Aisen, Reisa Sperling, Joshua D. Grill

**Affiliations:** 1 Alzheimer Therapeutic Research Institute, Keck School of Medicine, University of Southern California, San Diego; 2 Department of Neurology, Massachusetts General Hospital and Harvard Medical School, Boston, Massachusetts; 3 Department of Psychiatry, Massachusetts General Hospital and Harvard Medical School, Boston, Massachusetts; 4 Institute for Memory Impairments and Neurological Disorders, University of California Irvine; 5 Department of Neurology, Brigham and Women’s Hospital and Harvard Medical School, Boston, Massachusetts; 6 Department of Neurological Sciences, Rush Alzheimer’s Disease Center, Rush University, Chicago, Illinois; 7 Department of Psychiatry and Human Behavior, University of California Irvine; 8 Department of Neurobiology and Behavior, University of California Irvine

## Abstract

**Importance:**

Underrepresentation of many racial/ethnic groups in Alzheimer disease (AD) clinical trials limits generalizability of results and hinders opportunities to examine potential effect modification of candidate treatments.

**Objective:**

To examine racial and ethnic differences in recruitment methods and trial eligibility in a multisite preclinical AD trial.

**Design, Setting, and Participants:**

This cross-sectional study analyzed screening data from the Anti-Amyloid in Asymptomatic AD study, collected from April 2014 to December 2017. Participants were categorized into 5 mutually exclusive ethnic/racial groups (ie, Hispanic, Black, White, Asian, and other) using participant self-report. Data were analyzed from May through December 2020 and included 5945 cognitively unimpaired older adults between the ages of 65 and 85 years screened at North American study sites.

**Main Outcomes and Measures:**

Primary outcomes included recruitment sources, study eligibility, and ineligibility reasons. To assess the probability of trial eligibility, regression analyses were performed for the likelihood of being eligible after the first screening visit involving clinical and cognitive assessments.

**Results:**

Screening data were included for 5945 participants at North American sites (mean [SD] age, 71.7 [4.9] years; 3524 women [59.3%]; 5107 White [85.9%], 323 Black [5.4%], 261 Hispanic [4.4%], 112 Asian [1.9%], and 142 [2.4%] who reported race or ethnicity as other). Recruitment sources differed by race and ethnicity. While White participants were recruited through a variety of sources, site local recruitment efforts resulted in the majority of Black (218 [69.2%]), Hispanic (154 [59.7%]), and Asian (61 [55.5%]) participants. Participants from underrepresented groups had lower mean years of education (eg, mean [SD] years: Hispanic participants, 15.5 [3.2] years vs White participants, 16.7 [2.8] years) and more frequently were women (226 [70.0%] Black participants vs 1364 [58.5%] White participants), were unmarried (184 [56.9%] Black participants vs 1364 [26.7%] White participants), and had nonspousal study partners (237 [73.4%] Black participants vs 2147 [42.0%] White participants). They were more frequently excluded for failure to meet cognitive inclusion criteria (eg, screen failures by specific inclusion criteria: 147 [45.5%] Black participants vs 1338 [26.2%] White participants). Compared with White participants, Black (odds ratio [OR], 0.43; 95% CI, 0.34-0.54; *P* < .001), Hispanic (OR, 0.53; 95% CI, 0.41-0.69; *P* < .001), and Asian participants (OR, 0.56; 95% CI, 0.38-0.82; *P* = .003) were less likely to be eligible after screening visit 1.

**Conclusions and Relevance:**

Racial/ethnic groups differed in sources of recruitment, reasons for screen failure, and overall probability of eligibility in a preclinical AD trial. These results highlight the need for improved recruitment strategies and careful consideration of eligibility criteria when planning preclinical AD clinical trials.

## Key Points

**Question:**

Are there racial/ethnic differences associated with recruitment sources and reasons for ineligibility among preclinical Alzheimer disease clinical trial participants?

**Findings:**

In this cross-sectional study of screening data for 5945 participants from a preclinical Alzheimer disease trial, Black, Hispanic, and Asian participants were recruited from local efforts compared with White participants who were recruited from more distributed efforts. Adjusted analysis showed that underrepresented racial/ethnic communities were more likely to be ineligible after the first screening visit.

**Meaning:**

These findings suggest that there are racial and ethnic disparities in preclinical AD clinical trial enrollment that will require a comprehensive approach to study design and recruitment strategies to minimize disproportionate enrollment.

## Teaser

This cross-sectional study of screening data from a preclinical Alzheimer disease trial assesses differences in recruitment strategies and trial eligibility among racial and ethnic groups.

## Introduction

Randomized clinical trials are the criterion standard for testing the safety and efficacy of new therapies, devices, or procedures. It is imperative that trial samples represent the population who will use the intervention.^1,2,3^ Trials are also the last opportunity prior to formal approval to assess treatment effect modification in a controlled setting. Yet disparities persist in clinical trials, with several segments of the US population (eg, women and patients with fewer years of education and lower socioeconomic status) continuing to be underrepresented, particularly from communities of color.^4,5^

Several factors contribute to the lack of diversity in clinical research. Hispanic and non-White communities may have less access to expert care and diagnosis than their non-Hispanic White counterparts.^6,7,8^ They may face added logistical barriers to participation, may be less motivated or interested, or may lack the essential trust to consider enrolling.^9,10^ For participants who overcome the barriers to participation, trial screening criteria may disproportionately exclude them.^11^ In fact, in November 2020, the US Food and Drug Administration^12^ released formal guidance to promote representative recruitment of individuals with diverse demographic (ie, age, race, ethnicity, sex, gender identity, geographical location) and nondemographic (living status, comorbid conditions, disabilities) characteristics. Key among these recommendations was the need to carefully consider trial eligibility criteria and the risk of differential exclusion.

Alzheimer disease (AD) is a progressive neurodegenerative disease that is increasing in prevalence and cost at unsustainable levels.^13^ The risk and burden of AD are greater among African American/Black and Hispanic/Latino individuals compared with non-Hispanic White individuals.^14,15^ However, as in other areas of medical research, these and other racial and ethnic groups are underrepresented in AD clinical trials.^16,17,18^

Initial therapeutic efforts in AD focused on the dementia stage of the disease, with disappointing results. The discovery that disease biomarkers precede cognitive and functional impairment led to an era of secondary prevention clinical trials. These trials enroll participants with preclinical AD, who have evidence of AD biology on biomarker tests in the absence of cognitive impairment.^19,20^ Preclinical AD trials face difficultly in recruiting diverse participants,^21,22,23,24^ perhaps in part due to unique challenges in these novel studies.^25,26,27^ We examined the sources of recruitment and the reasons for screen failure among underrepresented racial and ethnic group participants in the first preclinical AD trial, the Anti-Amyloid treatment in Asymptomatic Alzheimer (A4) study.

## Methods

### Study Design and Selection

The A4 Study (ClinicalTrials.gov identifier: NCT02008357) is an ongoing 240-week, placebo-controlled, randomized Phase III clinical trial of an anti-Aβ monoclonal antibody in older adults with preclinical AD. The study design, details of the screening process and the characteristics of the participants have been previously described.^28^ Institutional review board approval was obtained at each of the performance sites. Written informed consent for use of prescreening data was provided by participants prior to any research activities being performed.

#### Study Recruitment

Centralized (by the coordinating center) and local (by the sites) recruitment strategies were employed. Case report forms (CRF) captured recruitment sources at enrollment. The form offered 5 categories: (1) internal source (site generated through databases, investigator clinical practices, or community outreach); (2) outside physician referrals (ie, from a physician who was not part of the trial and without formal relationship with the site); (3) organization referral (eg, Alzheimer Association, AARP, National Institute on Aging/National Institutes of Health); (4) paid advertisement; and (5) earned media, described as news or other non–paid content on television or radio, in print, or web-based. Sites indicated all appropriate sources for a given participant. For earned media, sites were requested to indicate “national,” “local,” or “don’t know” and provide additional details. Similarly, for paid advertisements and organizational referrals, additional specifics were requested. The CRF provided space for text to elaborate on the recruitment source regardless of the specific forced-choice responses. CRFs also captured participant demographics. This included participants’ age and years of education, as well as their race (American Indian or Alaskan Native, Asian, Native Hawaiian or other Pacific Islander, Black or African American, White, unknown or not reported) and ethnicity (Hispanic or Latino, not Hispanic or Latino, unknown or not reported).

#### Screening Process and Eligibility Criteria

The screening process included up to 5 visits completed within 90 days. An initial screening visit (ScV1) included relevant clinical assessments, cognitive testing, and *APOE* genotyping. Clinical and demographic requirements were assessed at ScV1, including: (1) between ages 65 and 85 years, (2) assessed to be cognitively unimpaired based on a Mini Mental Status Exam (MMSE) score between 25 and 30, Global Clinical Dementia (CDR) Rating Scale score of 0, and Logical Memory II score between 6 and 18, (3) stable medications, and (4) having a study partner with whom the participant had at least weekly contact and who was able to provide information on daily life and cognitive function annually. Initial screening eligibility criteria (ie, MMSE 27-30 and Logical Memory II score adjusted by education) were broadened early in the recruitment process in an effort to minimize screen failures among participants from underrepresented racial and ethnic groups. The study permitted participants with controlled hypertension, diabetes, hypercholesterolemia, mild-to-moderate small vessel cerebrovascular disease, and other common medical ailments. Exclusion criteria included: (1) current serious or unstable illness, (2) clinically significant laboratory abnormality, (3) clinically significant electrocardiogram, and (4) contraindications for magnetic resonance imaging studies.

Participants eligible after ScV1 underwent florbetapir Positron Emission Tomography (PET) at ScV2. Participants needed to demonstrate elevated brain amyloid on the florbetapir scan. Elevated amyloid was defined as a standardized uptake value ratio greater than 1.15 or between 1.0 and 1.15 with confirmed expert visual read of elevated amyloid.^28^ At ScV3, participants were provided their amyloid result (described as elevated or not elevated). Participants deemed eligible based on amyloid imaging continued on to a screening MRI visit (ScV4) and an optional lumbar puncture visit (ScV5) prior to being randomized.

### Statistical Analysis

For these analyses, only data from participants enrolled in North America were included. We assigned participants into 5 mutually exclusive racial/ethnic groups: Hispanic/Latino (Hispanic), non-Hispanic/Latino African American or Black (Black), non-Hispanic/Latino Asian (Asian), non-Hispanic/Latino Caucasian/White (White), and non-Hispanic/Latino “other racial group” (other). The other group included participants who self-reported as American Indian, Alaska Native, Native Hawaiian/Pacific Islander, indicated more than 1 race, or refused to answer, each of which represented a relatively small sample size. Participant characteristics at SCV1 were compared across groups using 2-sample *t* tests for continuous variables and χ^2^ tests for categorical variables.

Three investigators (M.B., M.R., and J.D.G.) reviewed recruitment source data for appropriate use of the CRF. Additional information provided was used to confirm and add recruitment sources as appropriate. Descriptive statistics (means with SDs and proportions) were used to describe the recruitment sources overall and by racial/ethnic subgroup. Racial/ethnic were compared using a Fisher exact test with Holm adjustment. For the category of “internal source,” we independently coded all additional comments by theme. A consensus-building exercise was used to arrive at the final set of themes. Since only a subset of CRFs included additional comments on specific sources, no quantification was performed.

Differences in the distribution of reasons for screen failure at ScV1 and ScV3 were compared using χ^2^ tests. Multivariable logistic regression analyses adjusted for age, sex, and education were used to compare the effect of race/ethnicity on eligibility after ScV1 and having elevated amyloid (for those who had PET scans). All analyses were conducted using the statistical software R version 3.6.2 (R Project for Statistical Computing).

## Results

### Participants and Recruitment Sources

Among 5945 participants enrolled at sites in North America (Table 1), 3524 reported as being women (59.3%). The mean (SD) age of participants was 71.7 (4.9) years and the mean education level was 16.5 (2.9) years. Most participants self-reported being White (5107 [85.9%]); 323 (5.4%) were Black, 261 (4.4%) were Hispanic, 112 (1.9%) were Asian, and 142 (2.4%) reported other race or ethnicity.

**Table 1.** Demographic Variables of Screened Participants by Racial/Ethnic Group

Characteristic	Race/ethnicity, No. (%)					*P* value^a^
	Hispanic (N = 261)	Black (N = 323)	White (N = 5107)	Asian (N = 112)	Other (N = 142)	
Age, mean (SD), y	71.8 (4.8)	71.3 (4.9)	71.7 (4.9)	72.5 (5.3)	71.5 (5.0)	.18
Education, mean (SD), y	15.5 (3.2)	15.4 (3.1)	16.7 (2.8)	16.9 (3.4)	16.6 (3.3)	<.001
Participant sex						
Female	163 (62.5)	226 (70.0)	2986 (58.5)	60 (53.6)	89 (62.7)	<.001
Male	98 (37.5)	97 (30.0)	2121 (41.5)	52 (46.4)	53 (37.3)	
Marital status						
Married	166 (63.6)	122 (37.8)	3655 (71.6)	80 (71.4)	86 (60.6)	<.001
Widowed	30 (11.5)	51 (15.8)	472 (9.2)	16 (14.3)	11 (7.7)	
Divorced	48 (18.4)	90 (27.9)	686 (13.4)	11 (9.8)	19 (13.4)	
Never married	9 (3.4)	43 (13.3)	206 (4.0)	5 (4.5)	9 (6.3)	
Unknown/other/NA	8 (3.1)	17 (5.3)	88 (1.7)	0	17 (12.0)	
Relationship to study partner						
Spouse	119 (45.6)	86 (26.6)	2960 (58.0)	55 (49.1)	70 (49.3)	<.001
Adult child	42 (16.1)	42 (13.0)	536 (10.5)	20 (17.9)	9 (6.3)	
Child-in-law	3 (1.1)	2 (0.6)	20 (0.4)	2 (1.8)	0	
Other relative	18 (6.9)	44 (13.6)	231 (4.5)	6 (5.4)	2 (1.4)	
Friend/companion	44 (16.9)	93 (28.8)	871 (17.1)	15 (13.4)	29 (20.4)	
Paid caregiver	0	3 (0.9)	1 (<0.1)	0	0	
Other	3 (1.1)	5 (1.5)	92 (1.8)	0	3 (2.1)	
Not applicable	32 (12.3)	48 (14.9)	396 (7.8)	14 (12.5)	29 (20.4)	
Study partner sex						
Female	151 (57.9)	181 (56.0)	2783 (54.5)	66 (58.9)	65 (45.8)	<.001
Male	110 (42.1)	142 (44.0)	2324 (45.5)	46 (41.1)	77 (54.2)	
Recruitment source^b^						
No.	258	315	5006	110	123	
Internal	154 (59.7)	218 (69.2)	2128 (42.5)	61 (55.5)	75 (61.0)	<.001
Outside physician	4 (1.5)	6 (1.9)	154 (3.1)	1 (0.9)	3 (2.4)	.77
Earned media – national	29 (11.2)	15 (4.8)	826 (16.5)	11 (10.0)	11 (8.9)	<.001
Earned media – local	41 (15.9)	38 (12.1)	1097 (21.9)	23 (20.9)	19 (15.4)	<.001
Earned media - unknown	9 (3.5)	7 (2.2)	186 (3.7)	4 (3.6)	5 (4.1)	.77
Paid advertising	10 (3.9)	17 (5.4)	362 (7.2)	12 (10.9)	4 (3.3)	.11
Organization	22 (8.5)	31 (9.8)	574 (11.5)	4 (3.6)	14 (11.4)	.12
*APOE* ε4 positive	53 (20.3)	79 (24.5)	1469 (28.8)	17 (15.2)	28 (19.7)	<.001

^a^ Kruskal-Wallis test for continuous variables and Fisher exact test for categorical variables.

^b^ Multiple sources of recruitment were possible, so the categories are not mutually exclusive. A Fisher exact test with Holm adjusted *P* values was used for comparisons.

The proportion of women was highest for Black participants (70.0%) and lowest for Asian participants (53.6%). White and Asian participants had a mean (SD) 16.7 (2.8) and 16.9 (3.4) years of education, compared with Hispanic and Black participants, who had 15.5 (3.2) and 15.4 (3.1) years, respectively. Black participants were more frequently not married (184 [56.9%]) compared with the other groups (range, 27.5%-33.3%). The types of study partners differed substantially among the groups, with White participants more often enrolling with a spouse (2960 [58.0%]) compared with Black (86 [26.6%]), Hispanic (119 [46%]), Asian (55 [49.1%]), or other participants (70 [49.3%]). Fewer Asian participants (17 [15.2%]) were carriers of the *APOE* ε4 allele compared with the other groups (range, 19.7%-28.8%).

### Recruitment Sources

Recruitment source data were available for 5812 participants (97.8%). Of 6175 recruitment records, 5900 records came through direct data capture and 275 records were identified through qualitative review. The most frequent sources of recruitment were through site internal sources (2636 [45.4%]) and earned media (2321 [39.9%]). Among referrals from earned media, 1218 (52.5%) resulted from local earned media and 892 (38.4%) from national earned media (with 211 [9.1%] referrals coming from unknown sources). Additional recruitment sources included organizational referrals (645 [11.1%]), paid advertising (405 [7.0%]) and outside referrals (168 [2.9%]). A detailed review of recruitment data indicated that the internal sources used by sites included other studies, community outreach, internal clinic referrals, mailings/brochures from the site, personal referrals by site physicians, and local registries. There were 226 (3.7%) unique earned media references resulting from an article that appeared in the Dear Abby syndicated column featuring the study’s principal investigator, Dr Reisa Sperling. Movie theater advertisements were indicated in 70 (1.1%) unique instances.

There were differences in referral sources among the racial/ethnic groups (Table 1). Site recruitment efforts were the primary source for Black (218 [69.2%]), Hispanic (154 [59.7%]), and Asian (61 [55.5%]) participants, followed by local earned media. White participants were recruited through more distributed sources, including site recruitment efforts (2128 [42.5%]), local earned media (1097 [21.9%]), national earned media (826 [16.5%]), and trusted organizations (574 [11.5%]).

### Screen Failures

Nearly one-third (1683 [28.3%]) of participants were excluded based on screening results at ScV1 (Table 2). The most frequent reasons for exclusion were based on the CDR (352 screen failures [20.9%]) and Logical Memory II scores (718 screen failures [42.7%]). Hispanic and non-White participants were excluded more frequently than were White participants (eg, 103 of 261 [39.5%] Hispanic participants vs 1338 of 5107 [26.2%] White participants) (Table 2). Exclusion based on MMSE criteria was most frequent for Hispanic participants (16 [6.1%]); Black participants were more frequently excluded based on screening results for CDR (41 [12.7%]) and Logical Memory II scores (62 [19.2%]). Examining the frequency of screen failure across cognitive criteria, the frequencies of exclusion were 26.1% for Hispanic (68 participants), 30.7% for Black (99 participants), 26.8% for Asian (30 participants), and 16.2% (825) for White participants. In models that controlled for covariates, Black (odds ratio [OR], 0.43; 95% CI, 0.34-0.54), Hispanic (OR, 0.53; 95% CI, 0.41-0.69), and Asian participants (OR, 0.56; 95% CI, 0.38-0.82) were less likely to be eligible to proceed after ScV1 compared with White participants as a referent group (Table 3).

**Table 2.** Frequency of Screen Failure by Criterion and Racial/Ethnic Group

Characteristic	Total, No.	Race/ethnicity, No. (%)				
		Hispanic	Black	White	Asian	Other
Total participants screened at ScV1	5945	261	323	5107	112	142
ScV1 screen failures^a^						
Screen failures by specific inclusion criteria^a^^,^^b^	1683 (28.3)	103 (39.5)	147 (45.5)	1338 (26.2)	43 (38.4)	52 (36.6)
MMSE score	100 (1.7)	16 (6.1)	15 (4.6)	60 (1.2)	4 (3.6)	5 (3.5)
Global CDR score at screening of 0	352 (5.9)	26 (10.0)	41 (12.7)	265 (5.2)	11 (9.8)	9 (6.3)
Logical Memory II score	718 (12.1)	42 (16.1)	62 (19.2)	570 (11.2)	21 (18.8)	23 (16.2)
≥1 of MMSE, CDR or Logical Memory II score	1056 (17.8)	68 (26.1)	99 (30.7)	825 (16.2)	30 (26.8)	34 (23.9)
Screen failures by specific exclusion criteria^a^^,^^b^						
Current serious or unstable illness	161 (2.7)	11 (4.2)	10 (3.1)	132 (3)	2 (2)	6 (4)
History of primary or recurrent malignant disease	59 (1.0)	2 (0.8)	2 (0.6)	54 (1.1)	0	1 (0.7)
Clinically significant ECG	106 (1.8)	4 (1.5)	7 (2.2)	93 (1.8)	1 (0.9)	1 (0.7)
History of immunological disorders	56 (0.9)	5 (1.9)	4 (1.2)	43 (0.8)	1 (0.9)	3 (2.1)
Clinically significant lab abnormalities	32 (0.5)	4 (1.5)	6 (1.9)	20 (0.4)	2 (1.8)	0
Total participants screened at ScV3^a^	3937 (66.2)	138 (52.9)	156 (48.3)	3507 (68.7)	62 (55.4)	74 (52.1)
ScV3 screen failures (PET scan showing nonelevated brain amyloid)^a^	2716 (45.7)	100 (38.3)	122 (37.8)	2393 (46.9)	53 (47.3)	48 (33.8)

Abbreviations: CDR, Clinical Dementia Rating; ECG, electrocardiogram; MMSE, Mini-Mental State Exam; ScV, screening visit.

^a^ Percentages are based on the total participants screened at ScV1 for a given race/ethnicity.

^b^ Limited to inclusion and exclusion criteria that accounted for at least 2% of the total screens within a group or over 50 participants overall.

**Table 3.** Multivariable Logistic Regression Model

	Model 1: Eligibility after ScV1 (N = 5735)		Model 2: Elevated amyloid (N = 3875)	
	Odds ratio (95% CI)	*P* value	Odds ratio (95% CI)	*P* value
Age at screening, y				
Women	0.96 (0.95-0.97)	<.001	1.05 (1.04-1.07)	<.001
Years of education	1.21 (1.08-1.36)	.001	1.00 (0.87-1.16)	>.99
Racial/ethnic group				
White (reference group)	1.05 (1.03-1.08)	<.001	0.99 (0.97-1.02)	.68
Hispanic	0.53 (0.41-0.69)	<.001	0.82 (0.55-1.19)	.31
Black	0.43 (0.34-0.54)	<.001	0.59 (0.39-0.86)	.008
Asian	0.56 (0.38-0.82)	.003	0.38 (0.17-0.73)	.007

Abbreviation: ScV, screening visit.

Among 3937 participants undergoing amyloid imaging, 2716 (69.0%) participants were excluded because they did not demonstrate elevated amyloid (Table 2). In a model examining the outcome of amyloid imaging eligibility, Black (OR, 0.59; 95% CI, 0.39-0.86) and Asian (OR, 0.38; 95% CI, 0.17-0.73) participants were less likely to be eligible compared with White participants as a referent group (Table 3).

## Discussion

Increasing the diversity of participants in preclinical AD trials will be essential to minimizing disparities in disease burden. The current results suggest that, even among the relatively small number of Hispanic and non-White participants recruited to the A4 trial, participants differed in their recruitment sources, their demographic and clinical characteristics, the reasons that they were excluded from participation, and their overall likelihood of being eligible for randomization. To the extent that the A4 Study is an accurate model of recruitment results for future preclinical AD trials, addressing each of these elements may be necessary to conduct truly inclusive studies of representative and generalizable samples (Table 4).

**Table 4.** Actionable Recommendations to Improve Recruitment and Enrollment of Underrepresented Racial and Ethnic Groups in Preclinical AD Trials

Recommendation	Rationale
Organizational structure	
Establish centralized prescreening databases	Prescreening databases permit real-time evaluation of outreach and screening efforts, allowing for the identification of the impact of centralized and local recruitment efforts while also assessing whether specific groups may be lost at varying levels of prescreening activities.
Establish a minimal data set for recruitment	Incorporating standardized data elements through the use of a minimal data set for recruitment ensures consistent data capture as it relates to race and ethnicity and may also enable collection of sociocultural factors (eg, education, occupation, socioeconomic status, neighborhood health variables), research attitudes, and other relevant constructs.
Provide earmarked funding for recruitment of participants from underrepresented groups	Line-item budgets do not typically offer differential reimbursement based on participant demographics. Offering specific support for sites to engage in diverse recruitment supports efforts to recruit underrepresented participants.
Select sites with diverse teams	Participants from underrepresented racial and ethnic groups may feel more comfortable communicating with and participating at sites with diverse study teams. Facilitate and reward sites for increasing the diversity of investigators.
Invest in community partnerships	Community-based organizations are often trusted gatekeepers. Providing funding to substantiate and strengthen relationships between sites and these organizations may enhance local recruitment of participants from underrepresented racial and ethnic groups. Engaging community advocates in study design and recruitment planning can ensure culturally sensitive designs. Identify participants from underrepresented groups who might serve as research ambassadors.
Study-specific approaches	
Develop a protocol-specific recruitment and retention plan	Comprehensive recruitment and retention study plans that prioritize diverse enrollment are essential to inclusivity and representation.
Develop specific recruitment strategies for unique underrepresented groups	Barriers to recruitment are likely to differ among unique communities. Unique strategies may be necessary to facilitate enrollment of underrepresented groups. This may require focus groups and market research to optimize recruitment messaging or may require more comprehensive strategies specific to groups.
Ensure that underrepresented groups are not disproportionately excluded by eligibility criteria	Broad inclusion criteria, adjusted for unique biological or cultural norms, may be necessary to optimally include participants from underrepresented groups. Performing data collection, especially cognitive testing, in non-English languages may be essential to ensuring inclusive enrollment.
Seek earned media opportunities to describe study recruitment needs	Earned media may increase awareness of the study through a trusted source. In particular, media stories through outlets serving underrepresented communities may offer promise.
Quantify site and central recruitment efforts and costs	Quantifying the costs associated with different recruitment strategies and cost per enrolled and randomized participant for each strategy can help future studies develop reasonable recruitment budgets.

The A4 study used a multitude of recruitment approaches. Overall, we found that internal site methods (such as referrals from other studies and community outreach) and earned media were the most frequent sources of participant recruitment. The study enjoyed unique coverage by national and local media. There were multiple national media stories that offered direct connections to study resources (ie, the call center phone number and/or the address of the study website). Among these, a Dear Abby column featuring the study principle investigator was particularly effective, generating more than 11 000 calls, more than 700 referrals to sites, and more than 200 screens. One A4 site leveraged national media coverage to identify potentially eligible participants who could be invited to local small group information sessions.^29^

In contrast to earned media, the A4 study ran numerous local and national advertisements that appeared to be less effective despite greater cost. Study advertisements included placements in national magazines, syndicated radio programs, and local newspapers. An exception to the ineffectiveness of advertisements was placements at local movie theaters. These advertisements were secured exclusively by sites, making their total number and cost unknown. Nonetheless, 70 unique screens were indicated to have come from this source.

Recruitment strategies varied in effectiveness among included racial/ethnic groups. Local site efforts were, by far, the most frequent sources of Black, Hispanic, and Asian participants, followed by local earned media. The ineffectiveness of centralized recruitment efforts, which included some specific advertising efforts, is consistent with the experiences of others^22,30,31^ and may indicate that trust and trustworthiness are critical to clinical trial recruitment and are not best established through paid advertising. Instead, community outreach and local earned media, both of which frequently involve site investigators and participants, may be more effective in engaging diverse communities.

Hispanic and non-White participants who were recruited to the A4 study differed from their White counterparts. They had lower education levels, making them more representative of the broader US population. They were more frequently women, unmarried, and had nonspousal study partners. These observations speak to important areas of need—men are underrepresented in preclinical AD trials, especially among Black and Hispanic communities. These observations may also alert investigators to potential sample biases brought on by the unique requirements of preclinical AD trials, namely the requirement of a study partner. This requirement has been reported to be a greater barrier for Black participants than their White counterparts^21^ and is concordant with observations in AD dementia trials, where Hispanic and non-White participants are more likely to have nonspouse study partners (eg, caregivers).^32^ Increased understanding of the attitudes of multiethnic groups toward the study partner requirement, as well as toward other unique aspects of participating in preclinical AD trials (eg, the requirement of amyloid biomarker disclosure^33^), should be prioritized to improve study designs and recruitment efforts.

Ensuring positive experiences of participants who overcome barriers to participation and enroll in studies may be essential to gaining and maintaining trust among underrepresented communities. The experience of enrolling in a trial only to be excluded based on eligibility criteria is often interpreted as rejection or being unwanted. We observed an alarmingly lower likelihood of eligibility among Black, Hispanic, and Asian participants compared with White participants. Eligibility criteria must be carefully selected to balance competing objectives of protecting participants from unnecessary risk, enroll individuals who are most likely to benefit from the study treatment, and produce research results that are generalizable to the larger population intended for treatment.^34^ In A4, Hispanic participants were more frequently excluded for MMSE criteria, Black participants for CDR criteria, and Hispanic, Black, and Asian participants for Logical Memory scores. Requirements on these elements were adjusted early in the trial in an effort to prevent differential exclusion by race and ethnicity. Whether eligible scores on these instruments could have been further adjusted for educational or cultural differences in normative scores is worth additional study and consideration in future trials. Improved tests that are more culturally sensitive or offer unbiased assessment are also needed.

Concordant with previous results from the A4 study,^28^ Black and Asian participants were less likely to demonstrate elevated amyloid compared with White participants when controlling for covariates among participants proceeding to ScV3 (a population differentially reduced in size from ScV1 for non-White participants). Hispanic participants also showed a trend toward less frequently demonstrating elevated amyloid. Relative to disease incidence estimates, the observation might be expected for Asian participants, but the results are surprising for Black and Hispanic participants.^14^ Recent observational studies have revealed noteworthy differences in AD biomarkers among racial groups.^35,36,37^ A more thorough understanding of the underpinnings of these disparities and whether they represent biological differences in disease manifestations or actual subgroup differences in the threshold between normal and abnormal will be essential to determining whether biomarker cutoffs should be universally applied to all participants in preclinical AD trials.

### Limitations

This study had several limitations. Although collected systematically through CRFs, data on recruitment sources were limited to forced-choice options that may not have perfectly accounted for the type or amount of effort required for participant recruitment. In particular, site expenditures, including spending on advertisements and staff effort, were not captured. Sites were supplemented financially for the specific purpose of increasing diverse recruitment, but how that support was used was not systematically tracked. We focused on overall trial results and did not account for site differences, for example in surrounding demographics. We lacked information on staff demographics, which has been shown by others to be important when recruiting diverse participants.^38,39^ Only a few sites offered participation in a non-English language, and this was limited to Spanish; this factor was not considered in these analyses. Clinical trials suffer consistently from sample bias. While this typically refers to the low representation of specific racial and ethnic groups, it may also reflect that those participants enrolled might not represent the larger population of that race or ethnicity.^40^ This risk is exacerbated by the relatively small sample sizes studied here, despite that they are among the largest samples of Black, Hispanic, and Asian participants in AD clinical trials.

Finally, our analyses were limited to self-reported constructs of race and ethnicity and failed to account for sociocultural factors that ultimately may prove more important than the categories used here. These constructs include socioeconomic status, literacy, acculturation, stigma, discrimination, neighborhood health metrics, and beliefs and attitudes about dementia.^41^ Few of these constructs were assessed in the A4 study. Future studies should assess these important variables to better elucidate potential predictors of trial eligibility, as well as potential effect modification of treatment safety and efficacy.

## Conclusions

The experiences from the A4 study may offer valuable insights investigators designing and conducting preclinical AD trials can use to optimize eligibility criteria and recruitment approaches (Table 4). Proactive and systematic approaches to implementing recruitment strategies, quantifying local and central recruitment efforts, and investing in local site resources and connections with community-based partners will be critical to recruitment of underrepresented communities. Once enrolled, broad inclusion criteria—potentially even adjusted for unique biological or cultural norms—may prevent disproportionate exclusion of these valuable participants.
